# Supplementary material for: Informing future research for carriage of multiresistant Gram-negative bacteria: problems with recruiting to an English stool sample community prevalence study
Source: BMJ Open. 2017 Dec 10;7(12):e017947. doi: 10.1136/bmjopen-2017-017947 (PMC5778273; doi:10.1136/bmjopen-2017-017947)
Supplement: Author's manuscript [file bmjopen-2017-017947.draft_revisions.pdf]

BMJ Open is committed to open peer review. As part of this commitment we make the peer review history of every article we publish publicly available.

When an article is published we post the peer reviewers' comments and the authors' responses online. We also post the versions of the paper that were used during peer review. These are the versions that the peer review comments apply to.

The versions of the paper that follow are the versions that were submitted during the peer review process. They are not the versions of record or the final published versions. They should not be cited or distributed as the published version of this manuscript.

BMJ Open is an open access journal and the full, final, typeset and author-corrected version of record of the manuscript is available on our site with no access controls, subscription charges or pay-per-view fees (http://bmjopen.bmj.com).

If you have any questions on BMJ Open's open peer review process please email editorial.bmjopen@bmj.com

Informing future research for carriage of multi-resistant gram negative bacteria: Problems with recruiting to an English stool sample community prevalence study.

Journal:	*BMJ Open*
Manuscript ID	bmjopen-2017-017947
Article Type:	Research
Date Submitted by the Author:	24-Jul-2017

Complete List of Authors:	Lecky, Donna; Public Health England Primary Care unit, Primary Care Unit<br>Nakiboneka-Ssenabulya, Deborah; Public Health England Primary Care unit, Primary Care Unit<br>Nichols, Tom; Public Health England Colindale<br>Hawkey, Peter; Heart of England NHS Foundation Trust, Public Health Laboratory<br>Thomas, Mike; University of Southampton Faculty of Health Sciences<br>Thomas, Helen Lucy; Public Health England Colindale<br>Chung, KeunTaik; Heart of England NHS Foundation Trust, Public Health Laboratory<br>Shabir, Sahida; Heart of England NHS Foundation Trust, Public Health Laboratory<br>Manzoor, Susan; Heart of England NHS Foundation Trust, Public Health Laboratory<br>Turner, Kim; Public Health England Primary Care unit, Primary Care Unit<br>Smith, Steve; Midlands & NW Bowel Cancer Screening Hub<br>Alvarez-Buylla, Adela; Heart of England NHS Foundation Trust, Public Health Laboratory<br>Xu McCrae, Li; Heart of England NHS Foundation Trust, Public Health Laboratory<br>McNulty, Cliodna; Public Health England Primary Care unit, Primary Care Unit

**Primary Subject Heading**:	Public health
Secondary Subject Heading:	Infectious diseases, Public health, Research methods
Keywords:	PRIMARY CARE, Public health < INFECTIOUS DISEASES, postal recruitment, Epidemiology < INFECTIOUS DISEASES, MICROBIOLOGY

SCHOLARONE™ Manuscripts

Informing future research for carriage of multi-resistant gram negative bacteria: Problems with recruiting to an English stool sample community prevalence study.

Lecky DM[1], Nakiboneka-Ssenabulya D[1], Nichols T[2], Hawkey P[3], Turner K[1], Thomas M[4], Thomas HL[5], Xu-McCrae L[3], Shabir S[3], Manzoor S[3], Alvarez-Buylla A[3], Smith S[6], Keun-Taik Chung[3] and McNulty CAM[1]*.

Donna Marie Lecky, Public Health England Primary Care Unit, Microbiology Department, Gloucester Royal Hospital, Great Western Road, Gloucester, Gloucestershire, GL1 3NN, UK. donna.lecky@phe.gov.uk

Deborah Nakiboneka-Ssenabulya, Public Health England Primary Care Unit, Microbiology Department, Gloucester Royal Hospital, Great Western Road, Gloucester, Gloucestershire, GL1 3NN, UK. debsenabulya@yahoo.com

Tom Nichols, Statistics, Modelling and Bioinformatics Department, Public Health England, 61 Colindale Avenue, London NW9 5EQ, UK. tom.nichols@phe.gov.uk

Peter Hawkey, Public Health Laboratory Birmingham, Heart of England NHS Foundation Trust, Birmingham B9 5SS. peter.hawkey@heartofengland.nhs.uk

Kim Turner, Public Health England Primary Care Unit, Microbiology Department, Gloucester Royal Hospital, Great Western Road, Gloucester, Gloucestershire, GL1 3NN, UK. kim.turner@phe.gov.uk

Mike Thomas, Primary Care, University of Southampton, Southampton, UK. D.M.Thomas@soton.ac.uk

Helen Lucy Thomas, TB Surveillance Unit, Public Health England. lucy.thomas@phe.gov.uk

Li Xu-McCrae, Public Health Laboratory Birmingham, Heart of England NHS Foundation Trust, Birmingham B9 5SS. li.xu@heartofengland.nhs.uk

Sahida Shabir, BMedSc, Public Health England, Bordesley Green E, Birmingham, England B9 5SS. Sahida.shabir@heartofengland.nhs.uk

Susan Manzoor, PhD, Public Health England, Bordesley Green E, Birmingham, England B9 5SS. Susan.manzoor@heartofengland.nhs.uk

Stephen Smith, PhD, Consultant Clinical Biochemist, University Hospitals of Coventry & Warwickshire NHS Trust, Midlands & NW Bowel Cancer Screening Hub, Hospital of St Cross, Barby Road, Rugby, CV22 5PX. steve.smith@uhcw.nhs.uk

Adela Alvarez-Buylla, PhD, Public Health England, Bordesley Green E, Birmingham, England B9 5SS. adela.buylla@heartofengland.nhs.uk

Keun-Taik Chung, Research Scientist, Public Health England, Bordesley Green E, Birmingham, England B9 5SS. Keun-taik.chung@hotmail.com

Cliodna Ann Miriam McNulty, Public Health England Primary Care Unit, Microbiology Department, Gloucester Royal Hospital, Great Western Road, Gloucester, Gloucestershire, GL1 3NN, UK. cliodna.mcnulty@phe.gov.uk

For peer review only - http://bmjopen.bmj.com/site/about/guidelines.xhtml

*Corresponding author: Prof Cliodna AM McNulty, cliodna.mcnulty@phe.gov.uk

For peer review only

For peer review only - http://bmjopen.bmj.com/site/about/guidelines.xhtml

Abstract

Objectives: This study aims to highlight problems with recruiting to an English stool sample community prevalence study .It was part of a larger cross sectional research to determine the risk factors for presence of Extended-Spectrum Beta-Lactamase and Carbapenemase Producing Coliforms (ESBLCPs) in stool samples of the asymptomatic general English population.

Setting: Four NHS Primary Care Trusts (PCTs) of England representing a different section of the population of England: Newham PCT; Heart of Birmingham Teaching PCT ; Shropshire County PCT; and Southampton City PCT.

Participants: Sixteen General Practices across the four PCTs were purposefully selected. After stratification of GP lists by age, ethnicity and antibiotic use, 58,337 randomly selected patients were sent a postal invitation.

Patients who had died, moved to a different surgery, were deemed too ill by their GP or hospitalised at the time of mailing were excluded.

Results: Stool and questionnaire returns varied by area, age, gender and ethnicity; the highest return rate of 27.3% was in Shropshire's over 60s; the lowest, 0.6%, was in Birmingham in the 18-39yr old age group. Whereas only 3.9 %(2,296) returned a completed questionnaire and stool sample, 94.9% of participants gave permission for their sample and data to be used in future research.

Conclusion: Researchers should consider the low stool specimen return rate and wide variation by ethnicity and age when planning future studies involving stool specimen collection. This is particularly pertinent if the study has no health benefit to participants. Further research is needed to explore how to improve recruitment in multi-cultural communities and in younger people.

For peer review only - http://bmjopen.bmj.com/site/about/guidelines.xhtml

Strengths

- This was a large multi-centre community based study that included adult participants of variable age groups, gender and ethnicities from four areas in England.
- By inviting a large number of patients from different ethnicities to participate, return rates are likely to be comparable in future studies.
- Recruiting patients in batches at each practice allowed us to compensate for the lower than expected return rate by increasing invited in cohorts with lower returns.
- Use of a stool collection instruction leaflet and a pre-packaged stool kit delivered to participant's homes may have aided compliance and stool returns.

Limitations

- Ethics permitted only anonymous patient information be removed from practices meaning researchers could not follow up with participants who did not respond to the initial invite; this is unfortunate as follow up phone calls and interaction with the research team encourage higher recruitment rates.

For peer review only - http://bmjopen.bmj.com/site/about/guidelines.xhtml

Background

Reports from the European Antimicrobial Resistance Surveillance Network (EARS-NET) data shows that multi-drug resistant *E.coli* now comprise 15% of invasive infections.[1] Researching gut carriage of multi-resistant bacteria in the asymptomatic population will help inform the need for control efforts as gut organisms are a source of gram negative infections. We do not know if prevalence research for gut carriage of antibiotic resistant organisms using postal stool samples is feasible, therefore understanding the challenges associated with obtaining postal stool samples is critical to the design of population-based research studies.

Recruitment of patients to research studies where they are asked to submit stool samples can be difficult, particularly when there is no obvious benefit to the participant. At community surveillance level the Bowel Cancer Screening Programme (BCSP), targeting adults over 60 years of age in England, found that stool specimen returns were 54% overall but lower among the Black and Minority Ethnic (BME) groups especially within the Asian population.[2] A general lack of opportunities to engage in research and cultural or religious practices have previously been highlighted as barriers to ethnic minority participation. [2-5] Problems with community recruitment can occur at different stages of the process e.g. obtaining patient lists, stratifying the data, obtaining consent, drop out following consent, etc.[6 7]but there is little information on population studies in asymptomatic individuals.

This paper aims to describe challenges faced when obtaining self-collected stool samples and self-administered questionnaires from healthy participants invited and recruited by post. It examines how stool return rate varied between different ethnic groups, age group, gender and the four National Health Service (NHS) Primary Care trusts (PCTs) selected. This research will inform future surveys using stool specimens.

For peer review only - http://bmjopen.bmj.com/site/about/guidelines.xhtml

Materials and Methods

PCT selection

Four NHS PCTs of England were selected non-randomly to represent a different section of the population of England: Newham PCT (London, urban, relatively high proportion of South Asian, Caribbean and African patients); Heart of Birmingham Teaching PCT (urban, very high proportion of South Asian patients); Shropshire County PCT (rural, very high proportion of White-British patients); and Southampton City PCT (semi-urban, high proportion of White-British and also a relatively high proportion of South Asian patients). Ethnicity data for each PCT was taken from Population Estimates by Ethnic Group in England[8] while the Index of Multiple Deprivation (IMD) for each practice was determined from online GP Practice Profiles.

GP Practice Selection

We worked with Primary Care Research Networks (PCRN) to facilitate recruitment of practices. All practices in a PCRN were invited by letter to participate. As ethnicity was a key criterion for patient selection, practices were excluded if they had not recorded ethnicity for at least 50% of their patients. Four or five practices that were willing to participate and were from the PCRN of each PCT were non-randomly selected to broadly represent each PCT with respect to ethnicity and deprivation. Overall sixteen practices were recruited to the study; three from Shropshire, four from Newham, five from Southampton and four from Birmingham.

Patient Selection

The study aimed to recruit 390 participants from each specific ethic group (Black, White, Asian, Mixed, Unknown) across the four PCTs. Patients in selected practices aged 18 years and above were stratified by a number of factors, including GP record of ethnicity, gender, age and antibiotic use in the previous year.

For peer review only - http://bmjopen.bmj.com/site/about/guidelines.xhtml

Patient screening by practice clinician

Patient lists were screened by a practice clinician to check suitability for inclusion. Excluded participants included those who died, moved to a different surgery, were deemed too ill by their GP or hospitalised at the time of mailing.

Patient invitation process (Figure 1)

Between November 2013 and October 2014, stratified lists were randomised and patients invited in order from these lists. A disproportionately large number of invites were sent to patients from those strata containing ethnic minority group patients (disproportionate stratified random sampling). Patients received an invitation letter containing a sentence in English, and in four of the most commonly used non-English languages spoken in that GP practice, inviting them to request a translation of the study information in their preferred language. Letters explained that

- The main study aimed to find out what things made some people more likely to carry different bacteria in the gut.
- If they agreed to participate, they would be asked to return a stool specimen and a short questionnaire about things that may affect bacteria in the gut such as antibiotic use, hospital visits, diet and travel.
- Information would be kept confidential.
- They could opt out of the study at any time.
- Participants would be given the option to receive either a £5 gift voucher or donate £5 towards research of the same topic on return of both the questionnaire and sample.

Invitation letters were sent in five different batches from each GP practice, with mail-outs at least one month apart to facilitate project administration. After each mail-out, stool returns were monitored and the number of invitation letters sent out in later mail-outs adjusted in the light of the return rate from earlier mail-outs. At some practices all patients within some strata were invited.

For peer review only - http://bmjopen.bmj.com/site/about/guidelines.xhtml

Stool sample kits

If patients were willing to participate they were asked to return a reply slip with their contact details in a pre-paid envelope. Those who returned a positive response reply slip to the invitation were then sent a study information sheet, stool collection kit and questionnaire. The stool sample collection kit had been designed with input from the general public.[9] Returning the questionnaire and stool sample was taken as implied consent for participation. In addition, willing patients were asked to give written consent to allow the study team to check their medical records for any details on the questionnaire which needed clarifying, and to save their stool sample for future research. The information sheet reiterated information in the invitation letter, and that the results would help the NHS improve the treatment and control of infections in their community and hospital. The stool collection kit was pre-labelled with their unique study ID and date of birth and contained a pair of plastic gloves, a sterile 30ml plastic stool collection pot, pictorial instructions[9], a spill proof stool pot transporter and a pre-paid biological specimen return envelope. Participants were not asked to make any dietary restrictions prior to taking a stool sample; neither were they asked to stop any on-going medication. Involvement in this study did not entail any visits to the practice or face-to-face contact with the researchers.

Participants were asked to return, by post, the questionnaire, consent form and self-collected stool sample to the research laboratory in the pre-paid addressed envelope which fitted into a normal post box. Study flyers at practice receptions, local newspapers and local radio were utilised to publicise the study. Towards the end of the stool collection period we stopped sending kits to respondents within the over 40 year age group, and of white ethnicity as we had reached sufficient numbers of stool samples in these groups. If willing participants did not return the stool sample kit, but provided their telephone number via the invite return slip, researchers made a reminder phone call to ask them to return their samples and questionnaires. To maximise returns these phone calls were made at different times of day. If necessary a further kit was provided.

For peer review only - http://bmjopen.bmj.com/site/about/guidelines.xhtml

Sample size

Previous research has showed that bla_{CTX-M} ESBLPE colonisation in diagnostic samples in Birmingham varied from 8.1% in Europeans to 22.8% in Middle East/South Asians.[10] Thus to have an 80% chance of finding a difference in faecal colonisation between different ethnic groups to be significant the 5% level, assuming the “true” colonisation percentages were 6% for Europeans and 12% for Asians, a total of 390 in each ethnic group across all 4 regions giving a total of 1560 participants overall was required. We assumed a 7% overall return rate, and therefore initially planned to send out 20,400 invites.

Data analysis

Of the 58,337 patients sent a postal invitation, the percentage that returned both a stool sample and a completed questionnaire was calculated – forthwith called stool return rate. We investigated how the stool return rate varied by ethnicity, age group, gender and PCT.

Of the patients sent a postal invitation and returning both a stool sample and a completed questionnaire we calculated the percentage choosing the £5 gift voucher rather than choosing £5 to be donated towards research on the same topic. If participants ticked both boxes for a £5 gift voucher and for £5 to be donated to research, it was assumed that the participant preferred a voucher. If participants ticked neither box they were excluded from the analysis of what choice they made.

Participants ticking neither box (for giving consent or not giving consent) for allowing us to access their GP notes or use their data for future research, were assumed to have not given their consent for these two actions.

Ethics

Ethical approval was obtained from Frenchay National Research Ethics Committee, REC reference 13/SW/0017, and local study approval sought from Primary Care Trusts (now

For peer review only

For peer review only - http://bmjopen.bmj.com/site/about/guidelines.xhtml

referred to as Clinical Commissioning Groups) from each of the 4 regions and individual GP practices.

Results

Recruitment

Sixteen practices were recruited to the study; three from Shropshire, four from Newham, five from Southampton and four from Birmingham. Stratifying by ethnicity proved difficult; over 350 ethnic variables were recorded, many of which were ambiguous as descriptions were commonly geographical areas, religions, language spoken and nationality. For the purposes of creating strata based on the GP record of ethnic group, this study created five groups; Asian, Black, Other/Mixed, Unknown and White. In total, we recruited 346 Asian patients, 186 Black patients, 1709 White patients and 53 mixed/other patients.

Sample returns (Figure 1)

We invited 58,337 patients to participate and 4,186 (7·2%) expressed interest. Stool collection kits were sent to 3,389 (5.8%); 2,388 (70.4%) returned a questionnaire and 2,430 (71.7%) returned a stool samples. Overall 2,296 (3.9%) returned a complete sample i.e. **both** a stool sample and completed questionnaire. This included 253 of 535 participants who gave their phone number and were reminded by phone.

Returns by PCT, age group, ethnic group and gender were as follows:

PCT: Complete sample return from invites was 8.6% (762/8,885) in Shropshire PCT, 1.6% (152/9,385) in Birmingham, 3% (583/20,087) in Newham and 4% (799/19,980) in Southampton.

Age group: Complete sample return from invites was 10% (994/9,960) from patients over 60 years, 4.7% (750/15,907) from 40-59 year olds and 1.7% (552/32,470) from under 40 year olds.

For peer review only - http://bmjopen.bmj.com/site/about/guidelines.xhtml

Ethnic group: Complete sample return from invites was 6.8% (1101/16,181) from White patients, 1.6% (296/18,502) from Asians, 4.1% (171/4,146) from Blacks, 3.7% (79/2,133) from other/mixed and 3.8% (650/17,225) from those of unknown ethnicity. All patients in Shropshire were assumed as being from a white ethnic group.

Gender: Complete sample returns from female invites was 4.8% (1,309/27,540) and 3.2% (987/30,797) from males.

Return rates by PCT, age group and ethnic group are illustrated in Figure 2. The highest return rate of 27.3% was in Shropshire's (predominately white) over 60 year olds; the lowest, 0.6%, was in Birmingham in the 18-39yr old age group.

Incentives: Of participants who returned a completed questionnaire together with a stool sample, 42% (942/2261) requested a £5 gift voucher, 57.4% (1319) opted to donate the £5 to research while 1.6% (35) did not indicate a preference. Overall 18-39 year olds (59%) preferred a high street voucher whereas the over 60yr olds (70%) preferred a donation to research; this was evident across all PCTs (Figure 3). Among Indian, Pakistani or Bangladeshi participants 60% preferred a £5 gift voucher while among White participants 38% preferred a £5 gift voucher. 61% of participants in Birmingham requested a voucher, 44% in Newham, 39% in Shropshire and 39% in Southampton.

Permissions: 94.9% (2178/2296) of participants who provided a stool and completed questionnaire gave consent for researchers to access their GP notes to clarify any details from the questionnaire. 94.9% (2180/2296) gave permission for their sample and data to be used in future research.

Discussion

Return rate

For peer review only - http://bmjopen.bmj.com/site/about/guidelines.xhtml

For peer review only

Participation rates in epidemiological studies especially population based studies have been declining over the years.[11] A US study found that the general public are divided on their willingness to participate in medical research trials; 46% surveyed via telephone said that they would participate in a study for a new treatment for a disease that concerns them, 25% were unwilling while 29% were undecided.[12]

The overall stool and questionnaire return rate from this study (3.9%) was lower than expected, resulting in difficulty in achieving our initial recruitment aim of 390 in each ethnic group; we had planned for a 7.6% return rate. Other research of a similar nature, a gut microbiome study, had a much higher return rate of 20%[3] however participants were all 55 – 69 year olds, were all female, received up to three follow up phone calls and had stool samples picked up by courier. We also found the return rate for females aged 60 or more was high but noted that this varied by ethnic group and that reminder phone calls proved particularly beneficial in increasing sample returns; 47% of those contacted returned the specimen.

Our highest return rate in the over 60yr olds (20%) is lower than for the Bowel Cancer Screening Programme (BCSP) pilot study in England and Scotland where the uptake was 57%-61.8% in patients 50 – 69 years.[13 14] A point of note is that our research had no personal benefit to the participant whilst the BCSP pilot study provided further cancer screening and treatment for those screened positive for bowel cancer.

Incentives

It has been previously noted that participation in research requires motivated individuals;[3] however the actual motivating factor varies. Offering study results as an incentive does not appear to increase recruitment. [15] Our study offered a £5 gift voucher or donating £5 to research as a potential motivation; of those who opted for a financial incentive, 18-39yr olds are more likely to want a voucher than over 60yr olds. Whilst we cannot say that a financial incentive was the main motivating factor for young participants, our findings do coincide with

For peer review only - http://bmjopen.bmj.com/site/about/guidelines.xhtml

other research,[7 16] suggesting that it may help facilitate recruitment in the younger age groups. However, when factoring this into a research plan, consideration should be given to the fact that the higher the financial incentive the more likely people are to agree to participate.[17]

Ethnicity

Lower uptake in BME groups compared to Whites has been reported elsewhere.[18] The global nature of transmission of multi-drug resistant bacteria[19] emphasizes the importance of ethnic minority participation in community surveillance of antimicrobial resistance. Some studies have found that BME groups are more willing to participate if they were approached directly and the research has direct relevance to them.[20] Language and cultural differences have been identified as barriers to recruitment of ethnic minority groups[4]. In each practice our information sheet has a sentence in the most common non English languages stating that the information could be provided in those languages; very few foreign language sheets were requested.

Future consent

Our low return rate suggests that those individuals that did participate may be more motivated than in the normal population, so it is unsurprising that 94.9% of our participants consented to allow researchers to access their GP notes and bank their sample and data for future research. Informing research for future generations has been cited as a motivating factor for consenting to bank samples.[21] Banking samples has been more commonly reported in genetic studies with blood or saliva samples where an over 90% consent rate has also been reported.[22]

Strengths and limitations

This was a large multi-centre community based study that included adult participants of variable age groups, gender and ethnicities from four areas in England. The majority of the

For peer review only - http://bmjopen.bmj.com/site/about/guidelines.xhtml

Asians in our study were from Birmingham and mostly spoke Urdu. Whilst we cannot categorically say that Asians from other areas of the Indian sub-continent would have similar low returns, other research involving stool returns has described uptake as *strikingly low* in ethnically diverse populations.[2] As we have invited a large number of patients from different ethnicities to participate, we feel that our return rates are likely to be comparable in future studies requesting stool samples from the general population.

Recruiting patients in batches at each practice was a strength of the sampling design because it allowed us to compensate for the lower than expected return rate by increasing invited in cohorts with lower returns.

Use of a stool collection instruction leaflet and a pre-packaged stool kit delivered to participant's homes may have aided compliance and stool returns.[23] In addition returning stool specimens by post had the advantage of reducing perceived embarrassment of returning a stool sample to a GP Practice receptionist.[9] Ethics permitted only anonymous patient information be removed from practices meaning researchers could not follow up with participants who did not respond to the initial invite; this is unfortunate as follow up phone calls and interaction with the research team encourage higher recruitment rates.[7 24] Patients received letters from their GP practice but were asked to send samples to a different location; this may have been confusing to some patients.

Conclusions

The low stool specimen return rate and it's wide variation by ethnicity and age, has implications for future studies that involve the collection of stool specimens from the general population and have no health benefit to their participants. Unless measures are taken to counteract this variation in the return rate, samples will tend to under-represent Asians and younger individuals. Furthermore research is needed to explore how to maximise stool return rates in research. Other forms of recruitment (other than postal recruitment) might be effective at increasing the return rate, however if postal is the recruitment method of choice

For peer review only - http://bmjopen.bmj.com/site/about/guidelines.xhtml

then reminder phone calls are recommended. Increasing the value of the gift voucher could be effective at increasing the return rate but obviously this increases the cost of the study and also risks introducing a new selection bias.

List of abbreviations

BCSP: Bowel Cancer Screening Programme

BME: Black and Minority Ethnic Groups

EARS-NET: European Antimicrobial Resistance Surveillance Network

IMD: Index of Multiple Deprivation

NHS: National Health Service

PCRN: Primary Care Research Network

PCT: Primary Care Trust

Ethics

Ethical approval for the study was obtained from the NRES Committee South West - Frenchay, Bristol, UK (13/SW/0017). The data we collected from GP practices was anonymous.

Competing Interests

All findings and observations are original unless otherwise acknowledged. The authors declared no competing interests.

Funding

This paper is independent research commissioned and funded by the Department of Health Policy Research Programme (Ref. 041/0038S). The views expressed in this publication are those of the author(s) and not necessarily those of the Department of Health.

For peer review only - http://bmjopen.bmj.com/site/about/guidelines.xhtml

For peer review only

Author Contributions

DL Project Manager Mar-Jul 2014 and from Apr 2015, was involved in data collection and data management, was a steering group member, and led the writing of the final manuscript.

DN-S Research Assistant was involved in ethics application, practice and participant recruitment, data collection and entry, and wrote the first draft of the manuscript.

TN Statistician: was grant co-applicant, involved in study design, practice and participant selection, data management, data analysis, was a steering group member, and contributed to the writing of the manuscript.

PH was grant co-applicant and involved in literature review, study design, questionnaire design, laboratory supervision, data interpretation, was a steering group member, and contributed to the writing of the manuscript.

KT Unit Administrator involved in participant recruitment and liaison, data collection and entry, and agreed the final manuscript.

MT was grant co-applicant and involved in study and questionnaire design, Steering Group member, Primary Care Lead, practice selection, and commented on the manuscript.

HLT Epidemiologist was grant co-applicant and involved in study and questionnaire design, data interpretation, was a steering group member, and commented on the manuscript.

LX-M Clinical Scientist was a grant co-applicant and involved in study design, laboratory supervision, supported laboratory data management, was a steering group member, and contributed to the writing of the manuscript.

SSh Research Scientist involved in laboratory work and data collection, and agreed the final manuscript.

SM Research Scientist involved in laboratory work and data collection, and agreed the final manuscript.

AA-B Research Scientist from was involved in laboratory work and data cleaning, and agreed the final manuscript.

For peer review only

SSm was grant co-applicant and involved in study design, was a steering group member, and commented on the manuscript.

K-TC Research Scientist involved in laboratory work, recording and data entry, and agreed the final manuscript.

CM Principal Investigator led the writing of the grant application and protocol, was involved in the literature review, contributed to the design of the questionnaire, led the project steering group, and led the writing of the manuscript.

For peer review only

Data Sharing

The relevant anonymised patient level data are available on reasonable request from the authors.

Acknowledgements

This independent research was commissioned and funded by the Department of Health Policy Research Programme. The views expressed in this publication are those of the author(s) and not necessarily those of the Department of Health. We are grateful for the support received from the Comprehensive Local Research Network, Clinical Research Network: Primary Care, Primary Care Unit Staff, Study GPs and Practice managers in the Birmingham area, Southampton, Shropshire and Newham.

For peer review only - http://bmjopen.bmj.com/site/about/guidelines.xhtml

References

1. Group IEARSNS. EARS-Net report, Quarters 1 - 4. Dublin 1, Ireland: Health Service Executive 2014.

2. von Wagner C, Baio G, Raine R, et al. Inequalities in participation in an organized national colorectal cancer screening programme: results from the first 2.6 million invitations in England. *Int J Epidemiol* 2011;40(3):712-18. doi: 10.1093/ije/dyr008

3. Feigelson HS, Bischoff K, Ardini MA, et al. Feasibility of self-collection of fecal specimens by randomly sampled women for health-related studies of the gut microbiome. *BMC Res Notes* 2014;7:204. doi: 10.1186/1756-0500-7-204

4. Sheikh A, Halani L, Bhopal R, et al. Facilitating the recruitment of minority ethnic people into research: qualitative case study of South Asians and asthma. *PLoS Med* 2009;6(10):e1000148. doi: 10.1371/journal.pmed.1000148

5. Adapting colorectal cancer screening for ethnic minority groups. Preconference 7th European Public Health Conference; 2014 20 November 2014; Glasgow, Scotland.

6. Galli L, Knight R, Robertson S, et al. Using marketing theory to inform strategies for recruitment: a recruitment optimisation model and the txt2stop experience. *Trials* 2014;22(15):182.

7. Watson JM, Torgerson DJ. Increasing recruitment to randomised trials: a review of randomised controlled trials. *BMC Med Res Methodol* 2006;6(1):1-9. doi: 10.1186/1471-2288-6-34

8. Office of National Statistics. PEEG, Current Estimates, Population Estimates by Ethnic Group Mid-2009 for Primary Care Organisations (experimental) (Excel sheet 739Kb) 2011.

For peer review only - http://bmjopen.bmj.com/site/about/guidelines.xhtml

9. Lecky DM, Hawking MK, McNulty CA, et al. Patients' perspectives on providing a stool sample to their GP: a qualitative study. *The British journal of general practice : the journal of the Royal College of General Practitioners* 2014;64(628):e684-93. doi: 10.3399/bjgp14X682261

10. Wickramasinghe NH, Xu L, Eustace A, et al. High community faecal carriage rates of CTX-M ESBL-producing Escherichia coli in a specific population group in Birmingham, UK. *The Journal of antimicrobial chemotherapy* 2012;67(5):1108-13.

11. Morton LM, Cahill J, Hartge P. Reporting participation in epidemiologic studies: a survey of practice. *Am J Epidemiol* 2006;163(3):197-203. doi: 10.1093/aje/kwj036

12. Trauth JM, Musa D, Siminoff L, et al. Public attitudes regarding willingness to participate in medical research studies. *J Health Soc Policy* 2000;12(2):23-43. doi: 10.1300/J045v12n02_02 [published Online First: 2001/02/24]

13. Logan RF, Patnick J, Nickerson C, et al. Outcomes of the Bowel Cancer Screening Programme (BCSP) in England after the first 1 million tests. *Gut* 2012;61(10):1439-46. doi: 10.1136/gutjnl-2011-300843

14. Moss SM, Campbell C, Melia J, et al. Performance measures in three rounds of the English bowel cancer screening pilot. *Gut* 2012;61(1):101-7. doi: 10.1136/gut.2010.236430

15. Cockayne S, Torgerson DJ. A randomised controlled trial to assess the effectiveness of offering study results as an incentive to increase response rates to postal questionnaires [ISRCTN26118436]. *BMC Med Res Methodol* 2005;5:34. doi: 10.1186/1471-2288-5-34

For peer review only - http://bmjopen.bmj.com/site/about/guidelines.xhtml

16. Martinson BC, Lazovich D, Lando HA, et al. Effectiveness of monetary incentives for recruiting adolescents to an intervention trial to reduce smoking. *Prev Med* 2000;31 doi: 10.1006/pmed.2000.0762

17. Treweek S, Lockhart P, Pitkethly M, et al. Methods to improve recruitment to randomised controlled trials: Cochrane systematic review and meta-analysis. *BMJ open* 2013;3(2) doi: 10.1136/bmjopen-2012-002360

18. Sinka K, Lacey M, Robertson C, et al. Acceptability and response to a postal survey using self-taken samples for HPV vaccine impact monitoring. *Sex Transm Infect* 2011;87(7):548-52. doi: 10.1136/sextrans-2011-050211

19. Hawkey PM. Multidrug-resistant Gram-negative bacteria: a product of globalization. *J Hosp Infect* 2015;89(4):241-7. doi: 10.1016/j.jhin.2015.01.008

20. Gill PS, Plumridge G, Khunti K, et al. Under-representation of minority ethnic groups in cardiovascular research: a semi-structured interview study. *Fam Pract* 2013;30(2):233-41. doi: 10.1093/fampra/cms054

21. Wong ML, Chia KS, Yam WM, et al. Willingness to donate blood samples for genetic research: the Asian Perspective. Rapid response (electronically published on 12 Oct 2001) to paper Stegmayr B, Asplund K. Informed consent for genetic research on blood stored for more than a decade. *BMJ* 2002;325:634-5.

22. Stegmayr B, Asplund K. Informed consent for genetic research on blood stored for more than a decade: a population based study. *BMJ* 2002;325(7365):634-35. doi: 10.1136/bmj.325.7365.634

23. Jones TF, Bulens SN, Gettner S, et al. Use of stool collection kits delivered to patients can improve confirmation of etiology in foodborne disease outbreaks. *Clin Infect Dis* 2004;39(10):1454-9. doi: 10.1086/425319 [published Online First: 2004/11/17]

For peer review only - http://bmjopen.bmj.com/site/about/guidelines.xhtml

24. de Wit NJ, Quartero AO, Zuithoff AP, et al. Participation and successful patient recruitment in primary care. *J Fam Pract* 2001;50(11):976.

For peer review only

For peer review only - http://bmjopen.bmj.com/site/about/guidelines.xhtml

STROBE Statement—Checklist of items that should be included in reports of ***cross-sectional studies***

	Item No	Recommendation	Reported	Page/Section
**Title and abstract**	1	(*a*) Indicate the study's design with a commonly used term in the title or the abstract	Yes	Abstract p3<br>Background p6
		(*b*) Provide in the abstract an informative and balanced summary of what was done and what was found	yes	p3
**Introduction**				
Background/rationale	2	Explain the scientific background and rationale for the investigation being reported	Yes	p5
Objectives	3	State specific objectives, including any prespecified hypotheses	Yes	p5
**Methods**				
Study design	4	Present key elements of study design early in the paper	Yes	Methods p6-7
Setting	5	Describe the setting, locations, and relevant dates, including periods of recruitment, exposure, follow-up, and data collection	Yes	Methods p6-8
Participants	6	(*a*) Give the eligibility criteria, and the sources and methods of selection of participants	Yes	Methods/patient selection p6-7
Variables	7	Clearly define all outcomes, exposures, predictors, potential confounders, and effect modifiers. Give diagnostic criteria, if applicable	Yes	Methods p6-8
Data sources/ measurement	8*	For each variable of interest, give sources of data and details of methods of assessment (measurement). Describe comparability of assessment methods if there is more than one group	Yes	Data analysis p9
Bias	9	Describe any efforts to address potential sources of bias	Yes	GP practice selection and patient selection p6
Study size	10	Explain how the study size was arrived at	Yes	Sample size p9
Quantitative variables	11	Explain how quantitative variables were handled in the analyses. If applicable, describe which groupings were chosen and why	Yes	Data analysis p9
Statistical methods	12	(*a*) Describe all statistical methods, including those used to control for confounding	NA	
		(*b*) Describe any methods used to examine subgroups and	NA	

For peer review only - http://bmjopen.bmj.com/site/about/guidelines.xhtml

		interactions		
		(*c*) Explain how missing data were addressed	NA	
		(*d*) If applicable, describe analytical methods taking account of sampling strategy	Yes	Data analysis p9
		(*e*) Describe any sensitivity analyses	NA	
**Results**				
Participants	13*	(a) Report numbers of individuals at each stage of study—eg numbers potentially eligible, examined for eligibility, confirmed eligible, included in the study, completing follow-up, and analysed	Yes	Recruitment p10
		(b) Give reasons for non-participation at each stage	NA	
		(c) Consider use of a flow diagram	Yes	Figure 1
Descriptive data	14*	(a) Give characteristics of study participants (eg demographic, clinical, social) and information on exposures and potential confounders	Yes	Sample returns p10
		(b) Indicate number of participants with missing data for each variable of interest	NA	
Outcome data	15*	Report numbers of outcome events or summary measures	Yes	Sample returns p10-11
Main results	16	(*a*) Give unadjusted estimates and, if applicable, confounder-adjusted estimates and their precision (eg, 95% confidence interval). Make clear which confounders were adjusted for and why they were included	NA	
		(*b*) Report category boundaries when continuous variables were categorized	NA	
		(*c*) If relevant, consider translating estimates of relative risk into absolute risk for a meaningful time period	NA	
Other analyses	17	Report other analyses done—eg analyses of subgroups and interactions, and sensitivity analyses	Yes	Results p11
**Discussion**				
Key results	18	Summarise key results with reference to study objectives	Yes	Discussion p11-13
Limitations	19	Discuss limitations of the study, taking into account sources of	Yes	Strengths and limitations p13-14

For peer review only

For peer review only - http://bmjopen.bmj.com/site/about/guidelines.xhtml

		potential bias or imprecision. Discuss both direction and magnitude of any potential bias		
Interpretation	20	Give a cautious overall interpretation of results considering objectives, limitations, multiplicity of analyses, results from similar studies, and other relevant evidence	Yes	Discussion/Conclusion p11-15
Generalisability	21	Discuss the generalisability (external validity) of the study results	Yes	Conclusion p14
**Other information**				
Funding	22	Give the source of funding and the role of the funders for the present study and, if applicable, for the original study on which the present article is based	Yes	Funding p15

*Give information separately for exposed and unexposed groups.

Note: An Explanation and Elaboration article discusses each checklist item and gives methodological background and published examples of transparent reporting. The STROBE checklist is best used in conjunction with this article (freely available on the Web sites of PLoS Medicine at http://www.plosmedicine.org/, Annals of Internal Medicine at http://www.annals.org/, and Epidemiology at http://www.epidem.com/). Information on the STROBE Initiative is available at www.strobe-statement.org.

For peer review only

BMJ Open

Informing future research for carriage of multi-resistant gram negative bacteria: Problems with recruiting to an English stool sample community prevalence study.

Journal:	*BMJ Open*
Manuscript ID	bmjopen-2017-017947.R1
Article Type:	Research
Date Submitted by the Author:	20-Oct-2017
Complete List of Authors:	Lecky, Donna; Public Health England, Primary Care Unit<br>Nakiboneka-Ssenabulya, Deborah; Public Health England, Primary Care Unit<br>Nichols, Tom; Public Health England, Statistics, Modelling and Bioinformatics Department<br>Hawkey, Peter; Heart of England NHS Foundation Trust, Public Health Laboratory<br>Turner, Kim; Public Health England, Primary Care Unit<br>Chung, Keun-Taik; Heart of England NHS Foundation Trust<br>Thomas, Mike; University of Southampton Faculty of Health Sciences<br>Thomas, Helen; Public Health England, TB Surveillance Unit<br>Xu McCrae, Li; Heart of England NHS Foundation Trust, Public Health Laboratory<br>Shabir, Sahida; Heart of England NHS Foundation Trust, Public Health Laboratory<br>Manzoor, Susan; Heart of England NHS Foundation Trust, Public Health Laboratory<br>Alvarez-Buylla, Adela; Heart of England NHS Foundation Trust<br>Smith, Steve; Midlands & NW Bowel Cancer Screening Hub<br>McNulty, Cliodna; Public Health England, Primary Care Unit
**Primary Subject Heading**:	Public health
Secondary Subject Heading:	Infectious diseases, Public health, Research methods
Keywords:	PRIMARY CARE, Public health < INFECTIOUS DISEASES, postal recruitment, Epidemiology < INFECTIOUS DISEASES, MICROBIOLOGY

SCHOLARONE™
Manuscripts

Informing future research for carriage of multi-resistant gram negative bacteria: Problems with recruiting to an English stool sample community prevalence study.

Lecky DM*, Nakiboneka-Ssenabulya D, Nichols T, Hawkey P, Turner K, Chung K-T, Thomas M, Thomas HL, Xu McCrae L, Shabir S, Manzoor S, Alvarez-Buylla A, Smith S, and McNulty C.

Donna M Lecky, Public Health England Primary Care Unit, Gloucester, UK. donna.lecky@phe.gov.uk

Deborah Nakiboneka-Ssenabulya, Public Health England Primary Care Unit Gloucester, UK. debsenabulya@yahoo.com

Tom Nichols, Public Health England, Statistics, Modelling and Bioinformatics Department, London, UK. tom.nichols@phe.gov.uk

Peter Hawkey, Heart of England NHS Foundation Trust, Public Health Laboratory Birmingham, UK. peter.hawkey@heartofengland.nhs.uk

Kim Turner, Public Health England, Primary Care Unit, Gloucester, UK. kim.turner@blueyonder.co.uk

Keun-Taik Chung, Heart of England NHS Foundation Trust, Public Health Laboratory Birmingham, UK. Keun-taik.chung@hotmail.com

Mike Thomas, University of Southampton Faculty of Health Sciences, Southampton, UK. D.M.Thomas@soton.ac.uk

Helen Lucy Thomas, Public Health England, TB Surveillance Unit, London, UK. lucy.thomas@phe.gov.uk

Li Xu McCrae, Heart of England NHS Foundation Trust, Public Health Laboratory Birmingham, UK. li.xu@heartofengland.nhs.uk

Sahida Shabir, Heart of England NHS Foundation Trust, Public Health Laboratory Birmingham, UK. Sahida.shabir@heartofengland.nhs.uk

Susan Manzoor, Heart of England NHS Foundation Trust, Public Health Laboratory Birmingham, UK. Susan.manzoor@heartofengland.nhs.uk

Adela Alvarez-Buylla, Heart of England NHS Foundation Trust, Public Health Laboratory Birmingham, UK. adela.buylla@heartofbirmingham.nhs.uk

Steve Smith, Midlands & NW Bowel Cancer Screening Hub, Coventry, UK. steve.smith@uhcw.nhs.uk

Cliodna McNulty, Public Health England Primary Care Unit Gloucester, UK. cliodna.mcnulty@phe.gov.uk

*Corresponding author: Dr Donna M Lecky, donna.lecky@phe.gov.uk

For peer review only - http://bmjopen.bmj.com/site/about/guidelines.xhtml

Abstract

Objectives: This study aims to highlight problems with recruiting to an English stool sample community prevalence study .It was part of a larger cross sectional research to determine the risk factors for presence of Extended-Spectrum Beta-Lactamase and Carbapenemase Producing Coliforms (ESBLCPs) in stool samples of the asymptomatic general English population.

Setting: Four NHS Primary Care Trusts (PCTs) of England representing a different section of the population of England: Newham PCT; Heart of Birmingham Teaching PCT ; Shropshire County PCT; and Southampton City PCT.

Participants: Sixteen General Practices across the four PCTs were purposefully selected. After stratification of GP lists by age, ethnicity and antibiotic use, 58,337 randomly selected patients were sent a postal invitation.

Patients who had died, moved to a different surgery, were deemed too ill by their GP or hospitalised at the time of mailing were excluded.

Results: Stool and questionnaire returns varied by area, age, gender and ethnicity; the highest return rate of 27.3% was in Shropshire's over 60s; the lowest, 0.6%, was in Birmingham in the 18-39yr old age group. Whereas only 3.9 %(2,296) returned a completed questionnaire and stool sample, 94.9% of participants gave permission for their sample and data to be used in future research.

Conclusion: Researchers should consider the low stool specimen return rate and wide variation by ethnicity and age when planning future studies involving stool specimen collection. This is particularly pertinent if the study has no health benefit to participants. Further research is needed to explore how to improve recruitment in multi-cultural communities and in younger people.

For peer review only - http://bmjopen.bmj.com/site/about/guidelines.xhtml

Strengths

- This was a large multi-centre community based study that included adult participants of variable age groups, gender and ethnicities from four areas in England.
- By inviting a large number of patients from different ethnicities to participate, return rates are likely to be comparable in future studies.
- Recruiting patients in batches at each practice allowed us to compensate for the lower than expected return rate by increasing invited in cohorts with lower returns.
- Use of a stool collection instruction leaflet and a pre-packaged stool kit delivered to participant's homes may have aided compliance and stool returns.

Limitations

- Ethics permitted only anonymous patient information be removed from practices meaning researchers could not follow up with participants who did not respond to the initial invite; this is unfortunate as follow up phone calls and interaction with the research team encourage higher recruitment rates.

For peer review only - http://bmjopen.bmj.com/site/about/guidelines.xhtml

Background

Reports from the European Antimicrobial Resistance Surveillance Network (EARS-NET) data shows that multi-drug resistant *E.coli* now comprise 15% of invasive infections.[1] Researching gut carriage of multi-resistant bacteria in the asymptomatic population will help inform the need for control efforts as gut organisms are a source of gram negative infections. We do not know if prevalence research for gut carriage of antibiotic resistant organisms using postal stool samples is feasible, therefore understanding the challenges associated with obtaining postal stool samples is critical to the design of population-based research studies.

Recruitment of patients to research studies where they are asked to submit stool samples can be difficult, particularly when there is no obvious benefit to the participant. At community surveillance level the Bowel Cancer Screening Programme (BCSP), targeting adults over 60 years of age in England, found that stool specimen returns were 54% overall but lower among the Black and Minority Ethnic (BME) groups especially within the Asian population.[2] A general lack of opportunities to engage in research and cultural or religious practices have previously been highlighted as barriers to ethnic minority participation. [2-5] Problems with community recruitment can occur at different stages of the process e.g. obtaining patient lists, stratifying the data, obtaining consent, drop out following consent, etc.[6 7]but there is little information on population studies in asymptomatic individuals.

This paper aims to describe challenges faced when obtaining self-collected stool samples and self-administered questionnaires from healthy participants invited and recruited by post. It examines how stool return rate varied between different ethnic groups, age group, gender and the four National Health Service (NHS) Primary Care trusts (PCTs) selected. This research will inform future surveys using stool specimens.

For peer review only - http://bmjopen.bmj.com/site/about/guidelines.xhtml

Materials and Methods

PCT selection

Four NHS PCTs of England were selected non-randomly to represent a different section of the population of England: Newham PCT (London, urban, relatively high proportion of South Asian, Caribbean and African patients); Heart of Birmingham Teaching PCT (urban, very high proportion of South Asian patients); Shropshire County PCT (rural, very high proportion of White-British patients); and Southampton City PCT (semi-urban, high proportion of White-British and also a relatively high proportion of South Asian patients). Ethnicity data for each PCT was taken from Population Estimates by Ethnic Group in England[8] while the Index of Multiple Deprivation (IMD) for each practice was determined from online GP Practice Profiles.

GP Practice Selection

We worked with Primary Care Research Networks (PCRN) to facilitate recruitment of practices. All practices in a PCRN were invited by letter to participate. As ethnicity was a key criterion for patient selection, practices were excluded if they had not recorded ethnicity for at least 50% of their patients. Four or five practices that were willing to participate and were from the PCRN of each PCT were non-randomly selected to broadly represent each PCT with respect to ethnicity and deprivation. Overall sixteen practices were recruited to the study; three from Shropshire, four from Newham, five from Southampton and four from Birmingham.

Patient Selection

The study aimed to recruit 390 participants from each specific ethic group (Black, White, Asian, Mixed, Unknown) across the four PCTs. Patients in selected practices aged 18 years and above were stratified by a number of factors, including GP record of ethnicity, gender, age and antibiotic use in the previous year.

For peer review only - http://bmjopen.bmj.com/site/about/guidelines.xhtml

For peer review only

Patient screening by practice clinician

Patient lists were screened by a practice clinician to check suitability for inclusion. Excluded participants included those who died, moved to a different surgery, were deemed too ill by their GP or hospitalised at the time of mailing.

Patient invitation process (Figure 1)

Between November 2013 and October 2014, stratified lists were randomised and patients invited in order from these lists. A disproportionately large number of invites were sent to patients from those strata containing ethnic minority group patients (disproportionate stratified random sampling). Patients received an invitation letter containing a sentence in English, and in four of the most commonly used non-English languages spoken in that GP practice, inviting them to request a translation of the study information in their preferred language. Letters explained that

- The main study aimed to find out what things made some people more likely to carry different bacteria in the gut.
- If they agreed to participate, they would be asked to return a stool specimen and a short questionnaire about things that may affect bacteria in the gut such as antibiotic use, hospital visits, diet and travel.
- Information would be kept confidential.
- They could opt out of the study at any time.
- Participants would be given the option to receive either a £5 gift voucher or donate £5 towards research of the same topic on return of both the questionnaire and sample.

Invitation letters were sent in five different batches from each GP practice, with mail-outs at least one month apart to facilitate project administration. After each mail-out, stool returns were monitored and the number of invitation letters sent out in later mail-outs adjusted in the light of the return rate from earlier mail-outs. At some practices all patients within some strata were invited.

For peer review only - http://bmjopen.bmj.com/site/about/guidelines.xhtml

Stool sample kits

If patients were willing to participate they were asked to return a reply slip with their contact details in a pre-paid envelope. Those who returned a positive response reply slip to the invitation were then sent a study information sheet, stool collection kit and questionnaire. The stool sample collection kit had been designed with input from the general public.[9] Returning the questionnaire and stool sample was taken as implied consent for participation. In addition, willing patients were asked to give written consent to allow the study team to check their medical records for any details on the questionnaire which needed clarifying, and to save their stool sample for future research. The information sheet reiterated information in the invitation letter, and that the results would help the NHS improve the treatment and control of infections in their community and hospital. The stool collection kit was pre-labelled with their unique study ID and date of birth and contained a pair of plastic gloves, a sterile 30ml plastic stool collection pot, pictorial instructions[9], a spill proof stool pot transporter and a pre-paid biological specimen return envelope. Participants were not asked to make any dietary restrictions prior to taking a stool sample; neither were they asked to stop any on-going medication. Involvement in this study did not entail any visits to the practice or face-to-face contact with the researchers.

Participants were asked to return, by post, the questionnaire, consent form and self-collected stool sample to the research laboratory in the pre-paid addressed envelope which fitted into a normal post box. Study flyers at practice receptions, local newspapers and local radio were utilised to publicise the study. If willing participants did not return the stool sample kit, but provided their telephone number via the invite return slip, researchers made a reminder phone call to ask them to return their samples and questionnaires. To maximise returns these phone calls were made at different times of day. If necessary a further kit was provided.

Sample size

For peer review only - http://bmjopen.bmj.com/site/about/guidelines.xhtml

Previous research has showed that *bla*$_{CTX-M}$ ESBLPE colonisation in diagnostic samples in Birmingham varied from 8.1% in Europeans to 22.8% in Middle East/South Asians.[10] Thus to have an 80% chance of finding a difference in faecal colonisation between different ethnic groups to be significant the 5% level, assuming the “true” colonisation percentages were 6% for Europeans and 12% for Asians, a total of 390 in each ethnic group across all 4 regions giving a total of 1560 participants overall was required. We assumed a 7% overall return rate, and therefore initially planned to send out 20,400 invites.

Data analysis

Of the 58,337 patients sent a postal invitation, the percentage that returned both a stool sample and a completed questionnaire was calculated – forthwith called stool return rate. We investigated how the stool return rate varied by ethnicity, age group, gender and PCT.

Of the patients sent a postal invitation and returning both a stool sample and a completed questionnaire we calculated the percentage choosing the £5 gift voucher rather than choosing £5 to be donated towards research on the same topic. If participants ticked both boxes for a £5 gift voucher and for £5 to be donated to research, it was assumed that the participant preferred a voucher. If participants ticked neither box they were excluded from the analysis of what choice they made.

Participants ticking neither box (for giving consent or not giving consent) for allowing us to access their GP notes or use their data for future research, were assumed to have not given their consent for these two actions.

Ethics

Ethical approval was obtained from Frenchay National Research Ethics Committee, REC reference 13/SW/0017, and local study approval sought from Primary Care Trusts (now referred to as Clinical Commissioning Groups) from each of the 4 regions and individual GP practices.

For peer review only

Results

Recruitment

Sixteen practices were recruited to the study; three from Shropshire, four from Newham, five from Southampton and four from Birmingham. Stratifying by ethnicity proved difficult; over 350 ethnic variables were recorded, many of which were ambiguous as descriptions were commonly geographical areas, religions, language spoken and nationality. For the purposes of creating strata based on the GP record of ethnic group, this study created five groups; Asian, Black, Other/Mixed, Unknown and White. In total, we recruited 346 Asian patients, 186 Black patients, 1709 White patients and 53 mixed/other patients.

Sample returns (Figure 1)

We invited 58,337 patients to participate and 4,186 (7·2%) expressed interest. Stool collection kits were sent to 3,389 (5.8%) as we stopped sending kits to respondents within the over 40 year age group, and of white ethnicity as when we sufficient numbers of stool samples in these groups; 2,388 (70.4%) returned a questionnaire and 2,430 (71.7%) returned a stool samples. Overall 2,296 (3.9%) returned a complete sample i.e. **both** a stool sample and completed questionnaire. This included 253 of 535 participants who gave their phone number and were reminded by phone. However, we did not reach our goal of obtain 390 samples from each of the 4 defined ethnic groups.

Returns by PCT, age group, ethnic group and gender were as follows:

PCT: Complete sample return from invites was 8.6% (762/8,885) in Shropshire PCT, 1.6% (152/9,385) in Birmingham, 2.9% (583/20,087) in Newham and 3.9% (799/19,980) in Southampton.

Age group: Complete sample return from invites was 9.9% (994/9,960) from patients over 60 years, 4.7% (750/15,907) from 40-59 year olds and 1.7% (552/32,470) from under 40 year olds.

For peer review only - http://bmjopen.bmj.com/site/about/guidelines.xhtml

Ethnic group: Complete sample return from invites was 6.8% (1101/16,181) from White patients, 1.6% (296/18,502) from Asians, 4.1% (171/4,146) from Blacks, 3.7% (79/2,133) from other/mixed and 3.8% (650/17,225) from those of unknown ethnicity. All patients in Shropshire were assumed as being from a white ethnic group.

Gender: Complete sample returns from female invites was 4.8% (1,309/27,540) and 3.2% (987/30,797) from males.

Return rates by PCT, age group and ethnic group are illustrated in Figure 2. The highest return rate of 27.3% was in Shropshire's (predominately white) over 60 year olds; the lowest, 0.6%, was in Birmingham in the 18-39yr old age group.

Incentives: Of participants who returned a completed questionnaire together with a stool sample, 41.6% (942/2261) requested a £5 gift voucher, 57.4% (1319) opted to donate the £5 to research while 1.6% (35) did not indicate a preference. Overall 18-39 year olds (58.5%) preferred a high street voucher whereas the over 60yr olds (69.3%) preferred a donation to research; this was evident across all PCTs (Figure 3). Among Indian, Pakistani or Bangladeshi participants 60.0% preferred a £5 gift voucher while among White participants 38.0% preferred a £5 gift voucher. 61.0% of participants in Birmingham requested a voucher, 43.9% in Newham, 38.7% in Shropshire and 39.1% in Southampton.

Permissions: 94.9% (2178/2296) of participants who provided a stool and completed questionnaire gave consent for researchers to access their GP notes to clarify any details from the questionnaire. 94.9% (2180/2296) gave permission for their sample and data to be used in future research.

Discussion

Return rate

For peer review only - http://bmjopen.bmj.com/site/about/guidelines.xhtml

Participation rates in epidemiological studies especially population based studies have been declining over the years.[11] A US study found that the general public are divided on their willingness to participate in medical research trials; 46% surveyed via telephone said that they would participate in a study for a new treatment for a disease that concerns them, 25% were unwilling while 29% were undecided.[12]

The overall stool and questionnaire return rate from this study (3.9%) was lower than expected, resulting in difficulty in achieving our initial recruitment aim of 390 in each ethnic group; we had planned for a 7.6% return rate. The nature of the sample collection i.e. faeces, may have contributed to this low return rate. Previous research examining why patients fail to return stool samples to their GP suggested that "the taboo associated with the 'dirtiness' of human faeces may be a key reason why some people lack the motivation to comply".[9] However, other research of a similar nature, a gut microbiome study, had a much higher return rate of 20%[3] however participants were all 55 – 69 year olds, were all female, received up to three follow up phone calls and had stool samples picked up by courier. We also found the return rate for females aged 60 or more was high but noted that this varied by ethnic group and that reminder phone calls proved particularly beneficial in increasing sample returns; 47% of those contacted returned the specimen.

Our highest return rate in the over 60yr olds (20%) is lower than for the Bowel Cancer Screening Programme (BCSP) pilot study in England and Scotland where the uptake was 57%-61.8% in patients 50 – 69 years.[13,14] A point of note is that our research had no personal benefit to the participant whilst the BCSP pilot study provided further cancer screening and treatment for those screened positive for bowel cancer.

Incentives

It has been previously noted that participation in research requires motivated individuals;[3] however the actual motivating factor varies. Offering study results as an incentive does not appear to increase recruitment. [15] Our study offered a £5 gift voucher or donating £5 to

For peer review only - http://bmjopen.bmj.com/site/about/guidelines.xhtml

research as a potential motivation; of those who opted for a financial incentive, 18-39yr olds are more likely to want a voucher than over 60yr olds. Whilst we cannot say that a financial incentive was the main motivating factor for young participants, our findings do coincide with other research,[7 16] suggesting that it may help facilitate recruitment in the younger age groups. However, when factoring this into a research plan, consideration should be given to the fact that the higher the financial incentive the more likely people are to agree to participate.[17]

Ethnicity

Lower uptake in BME groups compared to Whites has been reported elsewhere.[18] The global nature of transmission of multi-drug resistant bacteria[19] emphasizes the importance of ethnic minority participation in community surveillance of antimicrobial resistance. Some studies have found that BME groups are more willing to participate if they were approached directly and the research has direct relevance to them.[20] Language and cultural differences have been identified as barriers to recruitment of ethnic minority groups[4]. In each practice our information sheet has a sentence in the most common non English languages stating that the information could be provided in those languages; very few foreign language sheets were requested.

Future consent

Our low return rate suggests that those individuals that did participate may be more motivated than in the normal population, so it is unsurprising that 94.9% of our participants consented to allow researchers to access their GP notes and bank their sample and data for future research. Informing research for future generations has been cited as a motivating factor for consenting to bank samples.[21] Banking samples has been more commonly reported in genetic studies with blood or saliva samples where an over 90% consent rate has also been reported.[22]

For peer review only - http://bmjopen.bmj.com/site/about/guidelines.xhtml

For peer review only

Strengths and limitations

This was a large multi-centre community based study that included adult participants of variable age groups, gender and ethnicities from four areas in England. The majority of the Asians in our study were from Birmingham and mostly spoke Urdu. Whilst we cannot categorically say that Asians from other areas of the Indian sub-continent would have similar low returns, other research involving stool returns has described uptake as *strikingly low* in ethnically diverse populations.[2] As we have invited a large number of patients from different ethnicities to participate, we feel that our return rates are likely to be comparable in future studies requesting stool samples from the general population.

Recruiting patients in batches at each practice was a strength of the sampling design because it allowed us to compensate for the lower than expected return rate by increasing invited in cohorts with lower returns.

It could be argued that there are two different results that should be reported - willingness to participate is very low - 7%, but the return rate of samples is higher, 67.7% because 2,296/3,389 patients who were sent the collection kit returned complete sample and not the 3.9% (2,296/58,337) we report. However, we feel that by only reporting the return rate from those who expressed interest could be viewed as biased as we would be looking at those participants who are obviously interested in participating and would vastly underplay the amount of effort it requires to obtain a sufficient sample size from the general population.

Use of a stool collection instruction leaflet and a pre-packaged stool kit delivered to participant's homes may have aided compliance and stool returns.[23] In addition returning stool specimens by post had the advantage of reducing perceived embarrassment of returning a stool sample to a GP Practice receptionist.[9] Ethics permitted only anonymous patient information be removed from practices meaning researchers could not follow up with participants who did not respond to the initial invite; this is unfortunate as follow up phone calls and interaction with the research team encourage higher recruitment rates.[7 24] Patients

For peer review only - http://bmjopen.bmj.com/site/about/guidelines.xhtml

received letters from their GP practice but were asked to send samples to a different location; this may have been confusing to some patients.

Conclusions

The low stool specimen return rate and it's wide variation by ethnicity and age, has implications for future studies that involve the collection of stool specimens from the general population and have no health benefit to their participants. Unless measures are taken to counteract this variation in the return rate, samples will tend to under-represent Asians and younger individuals. Furthermore research is needed to explore how to maximise stool return rates in research. Other forms of recruitment (other than postal recruitment) might be effective at increasing the return rate, however if postal is the recruitment method of choice then reminder phone calls are recommended. Increasing the value of the gift voucher could be effective at increasing the return rate but obviously this increases the cost of the study and also risks introducing a new selection bias.

List of abbreviations

BCSP: Bowel Cancer Screening Programme

BME: Black and Minority Ethnic Groups

EARS-NET: European Antimicrobial Resistance Surveillance Network

IMD: Index of Multiple Deprivation

NHS: National Health Service

PCRN: Primary Care Research Network

PCT: Primary Care Trust

For peer review only - http://bmjopen.bmj.com/site/about/guidelines.xhtml

Ethics

Ethical approval for the study was obtained from the NRES Committee South West - Frenchay, Bristol, UK (13/SW/0017). The data we collected from GP practices was anonymous.

Competing Interests

All findings and observations are original unless otherwise acknowledged. The authors declared no competing interests.

Funding

This paper is independent research commissioned and funded by the Department of Health Policy Research Programme (Ref. 041/0038S). The views expressed in this publication are those of the author(s) and not necessarily those of the Department of Health.

Author Contributions

DL Project Manager Mar-Jul 2014 and from Apr 2015, was involved in data collection and data management, was a steering group member, and led the writing of the final manuscript.
DN-S Research Assistant was involved in ethics application, practice and participant recruitment, data collection and entry, and wrote the first draft of the manuscript.
TN Statistician: was grant co-applicant, involved in study design, practice and participant selection, data management, data analysis, was a steering group member, and contributed to the writing of the manuscript.
PH was grant co-applicant and involved in literature review, study design, questionnaire design, laboratory supervision, data interpretation, was a steering group member, and contributed to the writing of the manuscript.
KT Unit Administrator involved in participant recruitment and liaison, data collection and entry, and agreed the final manuscript.

For peer review only - http://bmjopen.bmj.com/site/about/guidelines.xhtml

MT was grant co-applicant and involved in study and questionnaire design, Steering Group member, Primary Care Lead, practice selection, and commented on the manuscript.

HLT Epidemiologist was grant co-applicant and involved in study and questionnaire design, data interpretation, was a steering group member, and commented on the manuscript.

LX-M Clinical Scientist was a grant co-applicant and involved in study design, laboratory supervision, supported laboratory data management, was a steering group member, and contributed to the writing of the manuscript.

SSh Research Scientist involved in laboratory work and data collection, and agreed the final manuscript.

SM Research Scientist involved in laboratory work and data collection, and agreed the final manuscript.

AA-B Research Scientist from was involved in laboratory work and data cleaning, and agreed the final manuscript.

SSm was grant co-applicant and involved in study design, was a steering group member, and commented on the manuscript.

K-TC Research Scientist involved in laboratory work, recording and data entry, and agreed the final manuscript.

CM Principal Investigator led the writing of the grant application and protocol, was involved in the literature review, contributed to the design of the questionnaire, led the project steering group, and led the writing of the manuscript.

Data Sharing

The relevant anonymised patient level data are available on reasonable request from the authors.

For peer review only - http://bmjopen.bmj.com/site/about/guidelines.xhtml

Acknowledgements

This independent research was commissioned and funded by the Department of Health Policy Research Programme. The views expressed in this publication are those of the author(s) and not necessarily those of the Department of Health. We are grateful for the support received from the Comprehensive Local Research Network, Clinical Research Network: Primary Care, Primary Care Unit Staff, Study GPs and Practice managers in the Birmingham area, Southampton, Shropshire and Newham.

For peer review only

For peer review only - http://bmjopen.bmj.com/site/about/guidelines.xhtml

References

1. Group IEARSNS. EARS-Net report, Quarters 1 - 4. Dublin 1, Ireland: Health Service Executive 2014.

2. von Wagner C, Baio G, Raine R, et al. Inequalities in participation in an organized national colorectal cancer screening programme: results from the first 2.6 million invitations in England. *Int J Epidemiol* 2011;40(3):712-18. doi: 10.1093/ije/dyr008

3. Feigelson HS, Bischoff K, Ardini MA, et al. Feasibility of self-collection of fecal specimens by randomly sampled women for health-related studies of the gut microbiome. *BMC Res Notes* 2014;7:204. doi: 10.1186/1756-0500-7-204

4. Sheikh A, Halani L, Bhopal R, et al. Facilitating the recruitment of minority ethnic people into research: qualitative case study of South Asians and asthma. *PLoS Med* 2009;6(10):e1000148. doi: 10.1371/journal.pmed.1000148

5. Adapting colorectal cancer screening for ethnic minority groups. Preconference 7th European Public Health Conference; 2014 20 November 2014; Glasgow, Scotland.

6. Galli L, Knight R, Robertson S, et al. Using marketing theory to inform strategies for recruitment: a recruitment optimisation model and the txt2stop experience. *Trials* 2014;22(15):182.

7. Watson JM, Torgerson DJ. Increasing recruitment to randomised trials: a review of randomised controlled trials. *BMC Med Res Methodol* 2006;6(1):1-9. doi: 10.1186/1471-2288-6-34

8. Office of National Statistics. PEEG, Current Estimates, Population Estimates by Ethnic Group Mid-2009 for Primary Care Organisations (experimental) (Excel sheet 739Kb) 2011.

For peer review only - http://bmjopen.bmj.com/site/about/guidelines.xhtml

9. Lecky DM, Hawking MK, McNulty CA, et al. Patients' perspectives on providing a stool sample to their GP: a qualitative study. *The British journal of general practice : the journal of the Royal College of General Practitioners* 2014;64(628):e684-93. doi: 10.3399/bjgp14X682261

10. Wickramasinghe NH, Xu L, Eustace A, et al. High community faecal carriage rates of CTX-M ESBL-producing Escherichia coli in a specific population group in Birmingham, UK. *The Journal of antimicrobial chemotherapy* 2012;67(5):1108-13.

11. Morton LM, Cahill J, Hartge P. Reporting participation in epidemiologic studies: a survey of practice. *Am J Epidemiol* 2006;163(3):197-203. doi: 10.1093/aje/kwj036

12. Trauth JM, Musa D, Siminoff L, et al. Public attitudes regarding willingness to participate in medical research studies. *J Health Soc Policy* 2000;12(2):23-43. doi: 10.1300/J045v12n02_02 [published Online First: 2001/02/24]

13. Logan RF, Patnick J, Nickerson C, et al. Outcomes of the Bowel Cancer Screening Programme (BCSP) in England after the first 1 million tests. *Gut* 2012;61(10):1439-46. doi: 10.1136/gutjnl-2011-300843

14. Moss SM, Campbell C, Melia J, et al. Performance measures in three rounds of the English bowel cancer screening pilot. *Gut* 2012;61(1):101-7. doi: 10.1136/gut.2010.236430

15. Cockayne S, Torgerson DJ. A randomised controlled trial to assess the effectiveness of offering study results as an incentive to increase response rates to postal questionnaires [ISRCTN26118436]. *BMC Med Res Methodol* 2005;5:34. doi: 10.1186/1471-2288-5-34

For peer review only - http://bmjopen.bmj.com/site/about/guidelines.xhtml

16. Martinson BC, Lazovich D, Lando HA, et al. Effectiveness of monetary incentives for recruiting adolescents to an intervention trial to reduce smoking. *Prev Med* 2000;31 doi: 10.1006/pmed.2000.0762

17. Treweek S, Lockhart P, Pitkethly M, et al. Methods to improve recruitment to randomised controlled trials: Cochrane systematic review and meta-analysis. *BMJ open* 2013;3(2) doi: 10.1136/bmjopen-2012-002360

18. Sinka K, Lacey M, Robertson C, et al. Acceptability and response to a postal survey using self-taken samples for HPV vaccine impact monitoring. *Sex Transm Infect* 2011;87(7):548-52. doi: 10.1136/sextrans-2011-050211

19. Hawkey PM. Multidrug-resistant Gram-negative bacteria: a product of globalization. *J Hosp Infect* 2015;89(4):241-7. doi: 10.1016/j.jhin.2015.01.008

20. Gill PS, Plumridge G, Khunti K, et al. Under-representation of minority ethnic groups in cardiovascular research: a semi-structured interview study. *Fam Pract* 2013;30(2):233-41. doi: 10.1093/fampra/cms054

21. Wong ML, Chia KS, Yam WM, et al. Willingness to donate blood samples for genetic research: the Asian Perspective. Rapid response (electronically published on 12 Oct 2001) to paper Stegmayr B, Asplund K. Informed consent for genetic research on blood stored for more than a decade. *BMJ* 2002;325:634-5.

22. Stegmayr B, Asplund K. Informed consent for genetic research on blood stored for more than a decade: a population based study. *BMJ* 2002;325(7365):634-35. doi: 10.1136/bmj.325.7365.634

23. Jones TF, Bulens SN, Gettner S, et al. Use of stool collection kits delivered to patients can improve confirmation of etiology in foodborne disease outbreaks. *Clin Infect Dis* 2004;39(10):1454-9. doi: 10.1086/425319 [published Online First: 2004/11/17]

For peer review only - http://bmjopen.bmj.com/site/about/guidelines.xhtml

24. de Wit NJ, Quartero AO, Zuithoff AP, et al. Participation and successful patient recruitment in primary care. *J Fam Pract* 2001;50(11):976.

For peer review only

For peer review only - http://bmjopen.bmj.com/site/about/guidelines.xhtml

Figure legend

Figure 1.Participant recruitment.

Figure 2. Participant demographics by stool return rate.

Figure 3. Incentive options for participants who returned a stool sample and completed questionnaire by age and PCT.

For peer review only

For peer review only - http://bmjopen.bmj.com/site/about/guidelines.xhtml

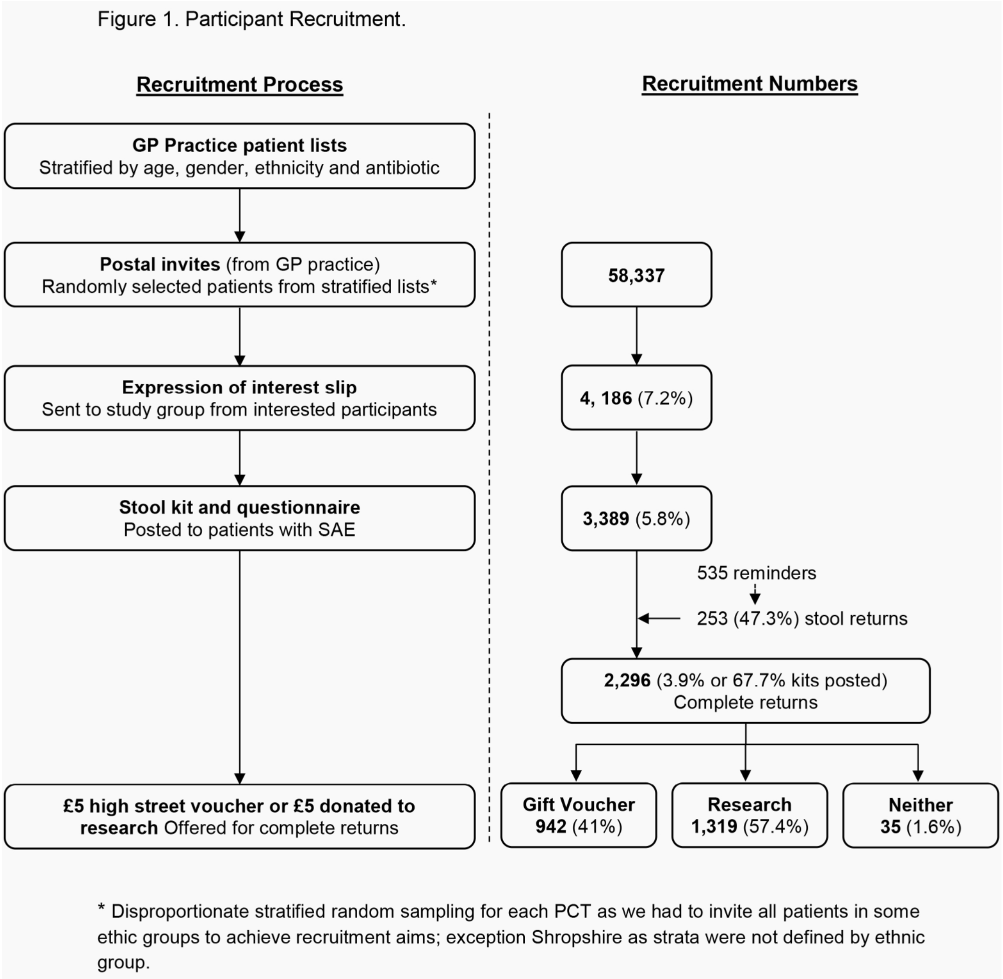

Figure 1.Participant recruitment

187x182mm (300 x 300 DPI)

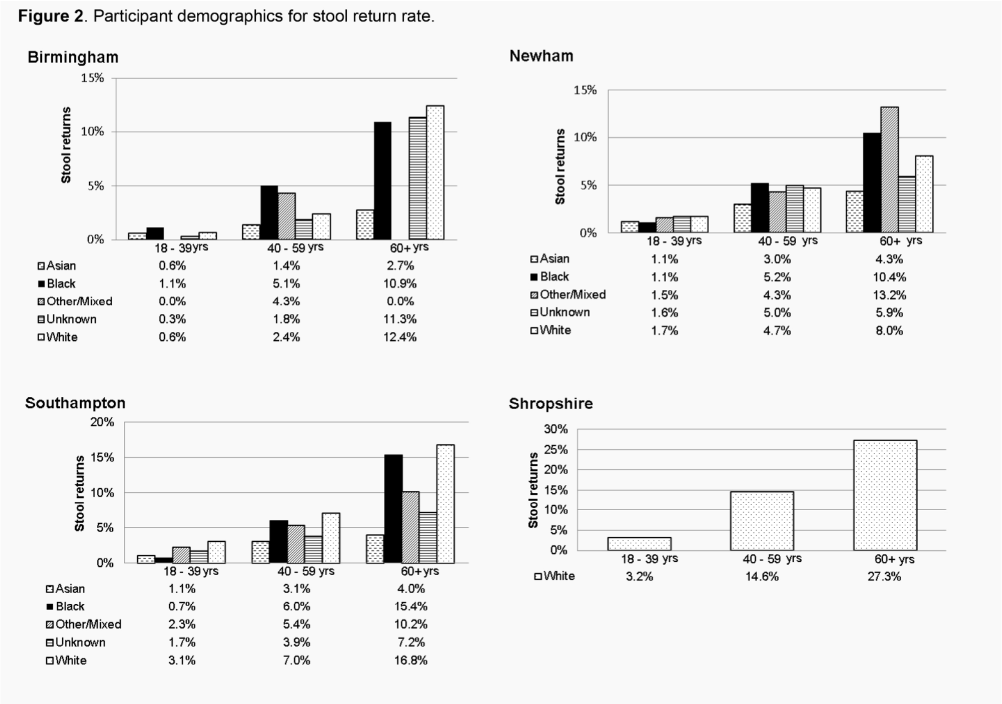

Figure 2. Participant demographics by stool return rate

230x161mm (300 x 300 DPI)

For peer review only - http://bmjopen.bmj.com/site/about/guidelines.xhtml

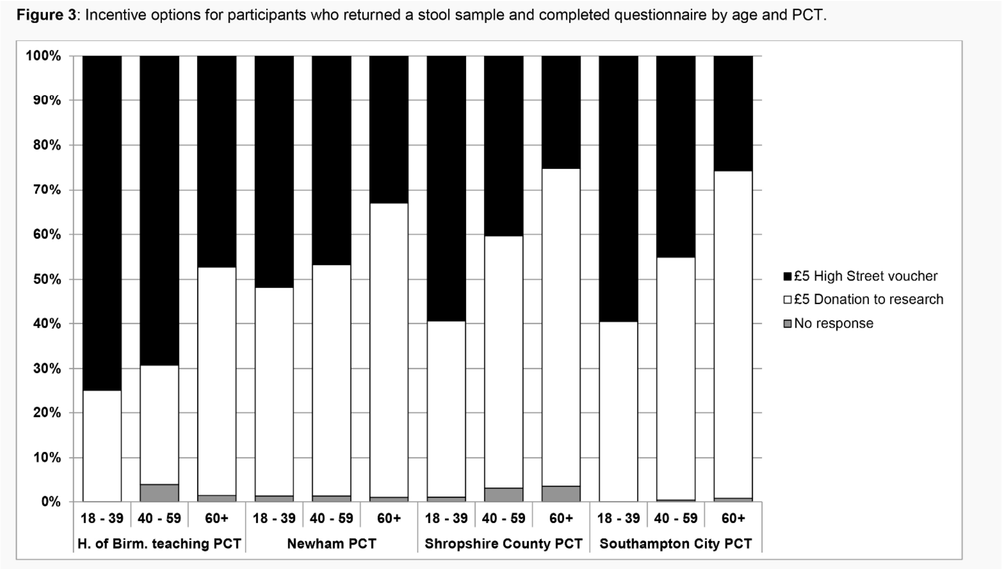

Figure 3. Incentive options for participants who returned a stool sample and completed questionnaire by age and PCT

251x143mm (300 x 300 DPI)

STROBE Statement—Checklist of items that should be included in reports of ***cross-sectional studies***

	Item No	Recommendation	Reported	Page/Section
**Title and abstract**	1	(*a*) Indicate the study's design with a commonly used term in the title or the abstract	Yes	Abstract p3 Background p6
		(*b*) Provide in the abstract an informative and balanced summary of what was done and what was found	yes	p3
**Introduction**				
Background/rationale	2	Explain the scientific background and rationale for the investigation being reported	Yes	p5
Objectives	3	State specific objectives, including any prespecified hypotheses	Yes	p5
**Methods**				
Study design	4	Present key elements of study design early in the paper	Yes	Methods p6-7
Setting	5	Describe the setting, locations, and relevant dates, including periods of recruitment, exposure, follow-up, and data collection	Yes	Methods p6-8
Participants	6	(*a*) Give the eligibility criteria, and the sources and methods of selection of participants	Yes	Methods/patient selection p6-7
Variables	7	Clearly define all outcomes, exposures, predictors, potential confounders, and effect modifiers. Give diagnostic criteria, if applicable	Yes	Methods p6-8
Data sources/ measurement	8*	For each variable of interest, give sources of data and details of methods of assessment (measurement). Describe comparability of assessment methods if there is more than one group	Yes	Data analysis p9
Bias	9	Describe any efforts to address potential sources of bias	Yes	GP practice selection and patient selection p6
Study size	10	Explain how the study size was arrived at	Yes	Sample size p9
Quantitative variables	11	Explain how quantitative variables were handled in the analyses. If applicable, describe which groupings were chosen and why	Yes	Data analysis p9
Statistical methods	12	(*a*) Describe all statistical methods, including those used to control for confounding	NA	
		(*b*) Describe any methods used to examine subgroups and	NA	

For peer review only - http://bmjopen.bmj.com/site/about/guidelines.xhtml

		interactions		
		(*c*) Explain how missing data were addressed	NA	
		(*d*) If applicable, describe analytical methods taking account of sampling strategy	Yes	Data analysis p9
		(*e*) Describe any sensitivity analyses	NA	
**Results**				
Participants	13*	(a) Report numbers of individuals at each stage of study—eg numbers potentially eligible, examined for eligibility, confirmed eligible, included in the study, completing follow-up, and analysed	Yes	Recruitment p10
		(b) Give reasons for non-participation at each stage	NA	
		(c) Consider use of a flow diagram	Yes	Figure 1
Descriptive data	14*	(a) Give characteristics of study participants (eg demographic, clinical, social) and information on exposures and potential confounders	Yes	Sample returns p10
		(b) Indicate number of participants with missing data for each variable of interest	NA	
Outcome data	15*	Report numbers of outcome events or summary measures	Yes	Sample returns p10-11
Main results	16	(*a*) Give unadjusted estimates and, if applicable, confounder-adjusted estimates and their precision (eg, 95% confidence interval). Make clear which confounders were adjusted for and why they were included	NA	
		(*b*) Report category boundaries when continuous variables were categorized	NA	
		(*c*) If relevant, consider translating estimates of relative risk into absolute risk for a meaningful time period	NA	
Other analyses	17	Report other analyses done—eg analyses of subgroups and interactions, and sensitivity analyses	Yes	Results p11
**Discussion**				
Key results	18	Summarise key results with reference to study objectives	Yes	Discussion p11-13
Limitations	19	Discuss limitations of the study, taking into account sources of	Yes	Strengths and limitations p13-14

For peer review only - http://bmjopen.bmj.com/site/about/guidelines.xhtml

		potential bias or imprecision. Discuss both direction and magnitude of any potential bias		
Interpretation	20	Give a cautious overall interpretation of results considering objectives, limitations, multiplicity of analyses, results from similar studies, and other relevant evidence	Yes	Discussion/Conclusion p11-15
Generalisability	21	Discuss the generalisability (external validity) of the study results	Yes	Conclusion p14
**Other information**				
Funding	22	Give the source of funding and the role of the funders for the present study and, if applicable, for the original study on which the present article is based	Yes	Funding p15

*Give information separately for exposed and unexposed groups.

Note: An Explanation and Elaboration article discusses each checklist item and gives methodological background and published examples of transparent reporting. The STROBE checklist is best used in conjunction with this article (freely available on the Web sites of PLoS Medicine at http://www.plosmedicine.org/, Annals of Internal Medicine at http://www.annals.org/, and Epidemiology at http://www.epidem.com/). Information on the STROBE Initiative is available at www.strobe-statement.org.

For peer review only

For peer review only - http://bmjopen.bmj.com/site/about/guidelines.xhtml
